# Non-contact smartphone-based fundus imaging compared to conventional fundus imaging: a low-cost alternative for retinopathy of prematurity screening and documentation

**DOI:** 10.1038/s41598-019-56155-x

**Published:** 2019-12-23

**Authors:** Maximilian W. M. Wintergerst, Michael Petrak, Jeany Q. Li, Petra P. Larsen, Moritz Berger, Frank G. Holz, Robert P. Finger, Tim U. Krohne

**Affiliations:** 1 Department of Ophthalmology, University of Bonn, Ernst-Abbe-Str. 2, 53127 Bonn, Germany; 2 Department of Medical Biometry, Informatics and Epidemiology, University Hospital Bonn, Sigmund-Freud-Str. 25, 53105 Bonn, Germany

**Keywords:** Retinopathy of prematurity, Medical imaging, Retinopathy of prematurity, Paediatric research

## Abstract

Retinopathy of prematurity (ROP) is a frequent cause of treatable childhood blindness. The current dependency of telemedicine-based ROP screening on cost-intensive equipment does not meet the needs in economically disadvantaged regions. Smartphone-based fundus imaging (SBFI) allows for affordable and mobile fundus examination and, therefore, could facilitate cost-effective telemedicine-based ROP screening in low-resources settings. We compared non-contact SBFI and conventional contact fundus imaging (CFI) in terms of feasibility for ROP screening and documentation. Twenty-six eyes were imaged with both SBFI and CFI. Field-of-view was smaller (ratio of diameters, 1:2.5), level of detail was equal, and examination time was longer for SBFI as compared to CFI (109.0 ± 57.8 vs. 75.9 ± 36.3 seconds, p < 0.01). Good agreement with clinical evaluation by indirect funduscopy was achieved for assessment of plus disease and ROP stage for both SBFI (squared Cohen’s kappa, 0.88 and 0.81, respectively) and CFI (0.86 and 0.93). Likewise, sensitivity/specificity for detection of plus disease and ROP was high for both SBFI (90%/100% and 88%/93%, respectively) and CFI (80%/100% and 100%/96%). SBFI is a non-contact and low-cost alternative to CFI for ROP screening and documentation that has the potential to considerably improve ROP care in middle- and low-resources settings.

## Introduction

Retinopathy of prematurity (ROP) is a frequent cause of treatable childhood visual impairment, particularly in middle-income countries, and is still becoming increasingly prevalent^1–4^. While highly developed countries have managed to reduce rates of ROP-related blindness with lessons learned from the first and second ROP epidemic by improvements in neonatal and ophthalmological patient care, moderately developed countries with human development index rankings between 31–100 struggle with proportions of childhood blindness due to ROP as high as 60%^5,6^. This results from a higher overall premature birth rate, inadequately monitored oxygen, survival of increasingly immature infants secondary to improved neonatal care, and a paucity of specialized medical personnel to provide ROP screening and treatment^5^.

World regions affected worst by this so-called “third epidemic” of ROP^1^ are Latin America and the former socialist economies of Eastern Europe, suffering from unsustainable situations such as only one ophthalmologist providing ROP service for a several million community^5,6^. Furthermore, India and China have the highest number of preterm births worldwide^7^ and the incidence of ROP has increased tremendously since neonatal care service has been introduced^6,8–10^. Due to financial constraints and scarcity of skilled personnel there is yet no comprehensive ROP screening and treatment program for most neonatal units in middle- and low-income countries^5^.

Against this background, the current dependency on ophthalmologists for ROP screening examinations is unlikely to meet the needs in many developing parts of the world^6^. Consequently, this will put tens of thousands of infants^7^ at risk of preventable blindness within the next decade secondary to insufficient ROP detection. Hence, there is a huge unmet need for novel ROP screening strategies, which are feasible, cost-effective and have high levels of sensitivity and specificity^5,6,11^. Possible solutions are the employment of paramedical staff such as pediatric nurses or medical technicians to provide first-level fundus examination for ROP screening^1,12^ as well as the use of telemedicine for remote ROP grading of digital fundus images^13^. Both have been shown to be successfully realizable^9–11,13–17^. However, conventional digital fundus imaging systems for ROP are still a high financial burden, particularly for health systems in middle- and low-resources settings and, hence, a considerable barrier for telemedicine-based ROP screening.

This might change with the advent of smartphone-based fundus imaging (SBFI), which allows for cheap and mobile fundus examination and documentation with the potential to revolutionize eye care especially in low-resources settings^18–20^. Although SBFI has already been successfully applied to diabetic retinopathy^21–26^ and glaucoma screening^27–29^, there is a dearth of studies of its application in ROP.

So far, only improvised techniques for indirect SBFI with a separate, hand-held funduscopy lens and few professional adapters have been tested in ROP^30–35^. Furthermore, no comparison with conventional fundus imaging systems used in ROP has been undertaken. To address these issues, we performed a study on the feasibility and accuracy of a non-contact indirect SBFI adapter for ROP documentation and its comparison with an established contact-based fundus imaging system for ROP.

## Methods

### Subject recruitment and clinical assessment

Infants eligible for ROP screening and follow-up were prospectively and consecutively recruited from the in- and out-patient ROP clinics at the Department of Ophthalmology, University of Bonn, Germany. Ethical approval was granted by the ethics committee of the University of Bonn (ID 205/17) and informed consent was obtained from the legal guardians of all study participants prior to study initiation. The study adhered to the tenets of the declaration of Helsinki. Exclusion criteria were the presence of retinal diseases other than ROP and optical media opacities, i.e. due to cataract, corneal opacity, or vitreous opacities. All eyes were assessed by indirect ophthalmoscopy for signs of ROP using topical anesthesia, scleral indentation, and external bulb rotation.

### Image acquisition

**Figure 1:**
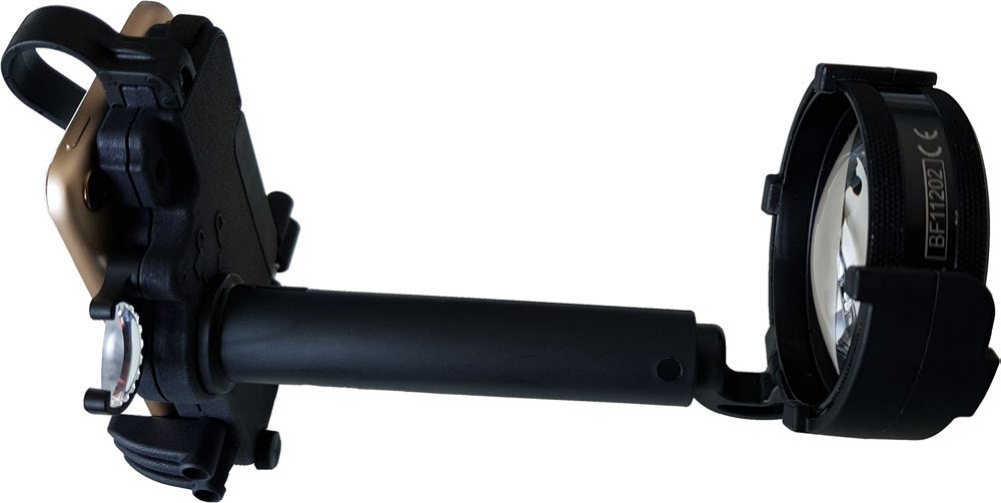
Smartphone-based fundus imaging device used for this study. The smartphone-based fundus imaging device was equipped with an iPod touch (sixth generation) and a Pan Retinal 2.2 for non-contact indirect smartphone-based fundus imaging.

Eyes were dilated and imaged with non-contact indirect SBFI using the Paxos Scope adapter (Fig. 1, version from 2016, DigiSight Technologies Inc./Verana Health Inc., San Francisco, California, USA) equipped with an iPod touch (6^th^ generation, Apple Inc., Cupertino, California, USA) and a Pan Retinal 2.2 or 40D lens (Volk Optical Inc., Mentor, Ohio, USA). Images acquired with the Pan Retinal 2.2 lens were used for comparison with indirect ophthalmoscopy. Control images were taken using the RetCam Shuttle (Natus Medical Inc., Pleasanton, California, USA) with a 130° lens. With both devices, imaging was performed in video mode with subsequent extraction of single images for analysis. SBFI was performed by the same examiner in all patients (MWMW) and conventional fundus imaging by two examiners (TUK or MP). Imaging protocol included examination of the posterior pole and at least the temporal part of the periphery. Scleral indentation and external bulb rotation were applied when necessary. All examinations for each eye were performed on the same day, under topical anesthesia, and in a darkened room.

### Image analysis

Fundus field-of-view was compared between SBFI and conventional fundus imaging. Ratios of field-of-view diameters and areas were calculated. Images were randomized and graded by two masked ophthalmologists experienced in ROP screening (TUK and MP) for stage and zone of ROP^36^, plus disease (on an eye-based method^37^ with a 3 step scale of “no plus disease”, “pre-plus disease”, and “plus disease”) and certainty of each evaluation on a 5 step scale (1 = unsure, 2 = rather unsure, 3 = moderately sure, 4 = rather sure, 5 = sure). Reason for uncertainty was documented in case of a certainty grading of 4 or less and coded on a nominal scale consisting of “fundus area examined too small”, “image blurred”, “overexposure”, and “grading inconclusive albeit good image quality”. All grading was performed on 23 inch monitors. If needed, fundus images were optimized for brightness and contrast in a post processing step (only entire images, no selective adjustments). Examination time was assessed using the recorded examination videos.

### Statistical analysis

Statistical analysis was performed using R software (R: A Language and Environment for Statistical Computing, R Core Team, R Foundation for Statistical Computing, Vienna, Austria, version 3.4.0, 2017). Weighted Cohen’s kappa values were calculated to assess agreement of plus disease and ROP grading with evaluation by indirect ophthalmoscopy and sensitivity/specificity to detect plus disease and ROP. Indirect ophthalmoscopy was used as the reference standard. Certainty of plus disease and ROP grading were compared between SBFI and conventional fundus imaging by Mann-Whitney-U-Test. Inter-rater reliability for plus disease and ROP grading were assessed using weighted Cohen’s kappa. Examination time was compared by Student’s t-test after normality testing by Shapiro-Wilk test.

## Results

### Demographics and clinical characteristics

Twenty-six eyes from 14 infants were included in the study. Demographics of the cohort are summarized in Table 1.

**Table 1:** Demographics.

		Mean ± SD or n (%)
Gestational age	mean (weeks)	25.9 ± 2.2
	range (weeks)	23.0–30.3
Birth weight	mean (grams)	779 ± 195
	range (grams)	580–1285
Postmenstrual age at examination	mean (weeks)	42.8 ± 9.2
	range (weeks)	29.0–67.4
Sex	Male	13 (50%)
	Female	13 (50%)
Plus disease	No plus disease	16 (61.5%)
	Pre-plus disease	7 (27%)
	Plus disease	3 (11.5%)
ROP stage	No ROP	14 (54%)
	Stage 1	1 (4%)
	Stage 2	6 (23%)
	Stage 3	5 (19%)
	Stage 4	0
	Stage 5	0
ROP zone	Zone I	2 (16.6%)
	Zone II	8 (66.6%)
	Zone III	2 (16.6%)

ROP = retinopathy of prematurity; SD = standard deviation.

### Evaluation of field-of-view and examination time

**Figure 2:**
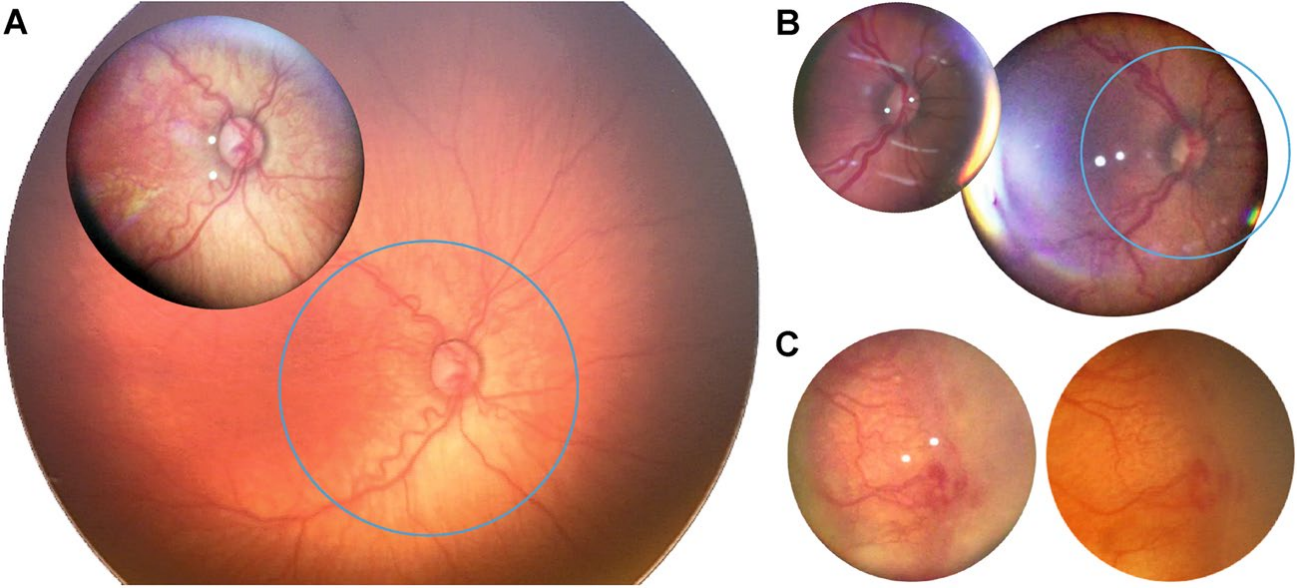
Comparison of field-of-view and image detail. (**A**) Smartphone-based fundus imaging (SBFI) with a Pan Retinal 2.2 lens (**A**, foreground) and conventional fundus imaging (**A**, background) were performed in the same eye. The blue circle represents the exact field-of-view covered by SBFI. (**B**) Comparison of SBFI with the Pan Retinal 2.2 lens (**B**, left) and the 40D lens (**B**, right). The blue circle represents the exact field-of-view covered by the Pan Retinal 2.2 lens. (**C**) The same pathology was documented with SBFI (**C**, left) and conventional fundus imaging (**C**, right). For comparative reasons only the image section from the conventional fundus image that corresponds exactly to the fundus area covered by SBFI is shown.

**Figure 3:**
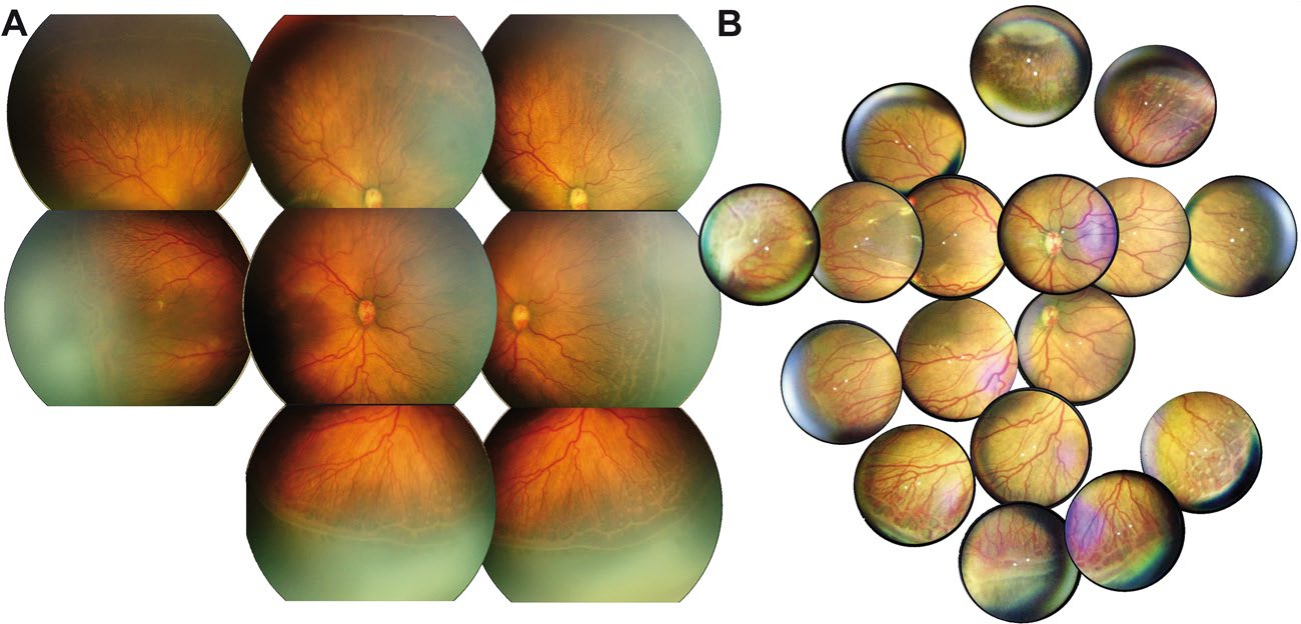
Comparison of composite fundus images. Conventional fundus imaging **(A**) and smartphone-based fundus imaging (**B)** were performed in the same eye under general anesthesia. A wide-field-montage was created to give an impression of the dynamic field-of-view during live examination.

The Pan Retinal 2.2 lens utilized in SBFI corresponds to a 56° field-of-view, whereas conventional fundus imaging was performed using a 130° field-of-view lens. A comparison of achieved field-of-view in premature infants is displayed in Fig. 2A. Ratios are 1: 2.5 for image diameters and 1: 5.7 for image areas. Although the field-of-view was smaller with SBFI, it was possible to examine a large area of the fundus including the periphery (Fig. 3). Due to optical principles, actual field-of-view depends on the size of the eye and hence gestational age^38^, with more mature eyes allowing for greater field-of-view (e.g. SBFI in an 48-weeks-old infant in Fig. 2A, compared with SBFI in an 36-weeks-old infant in Fig. 2B).

Indirect SBFI with the utilized adapter is also possible with other lenses than the Pan Retinal 2.2, e.g. with the 40D lens, which conveys a bigger field-of-view (Fig. 2B). Ratios are 1: 1.44 for image diameter and 1: 2.07 for image areas. However, image quality with the 40D lens appeared to be reduced (reduced image sharpness and increased reflexes, Fig. 2B), and imaging more challenging because the imaging position with minimal reflexes is harder to maintain. Therefore, SBFI in the further study was performed using the Pan Retinal 2.2 lens.

Examination time was significantly longer in SBFI than in conventional fundus imaging (mean ± standard deviation for SBFI and conventional fundus imaging, respectively: 109 ± 57.8 and 75.9 ± 36.3 seconds, p-value = 0.005, mean difference: − 33.1 seconds, 95% confidence interval: −11.0 to −55.1 seconds).

### Evaluation of plus disease and retinopathy of prematurity

**Figure 4:**
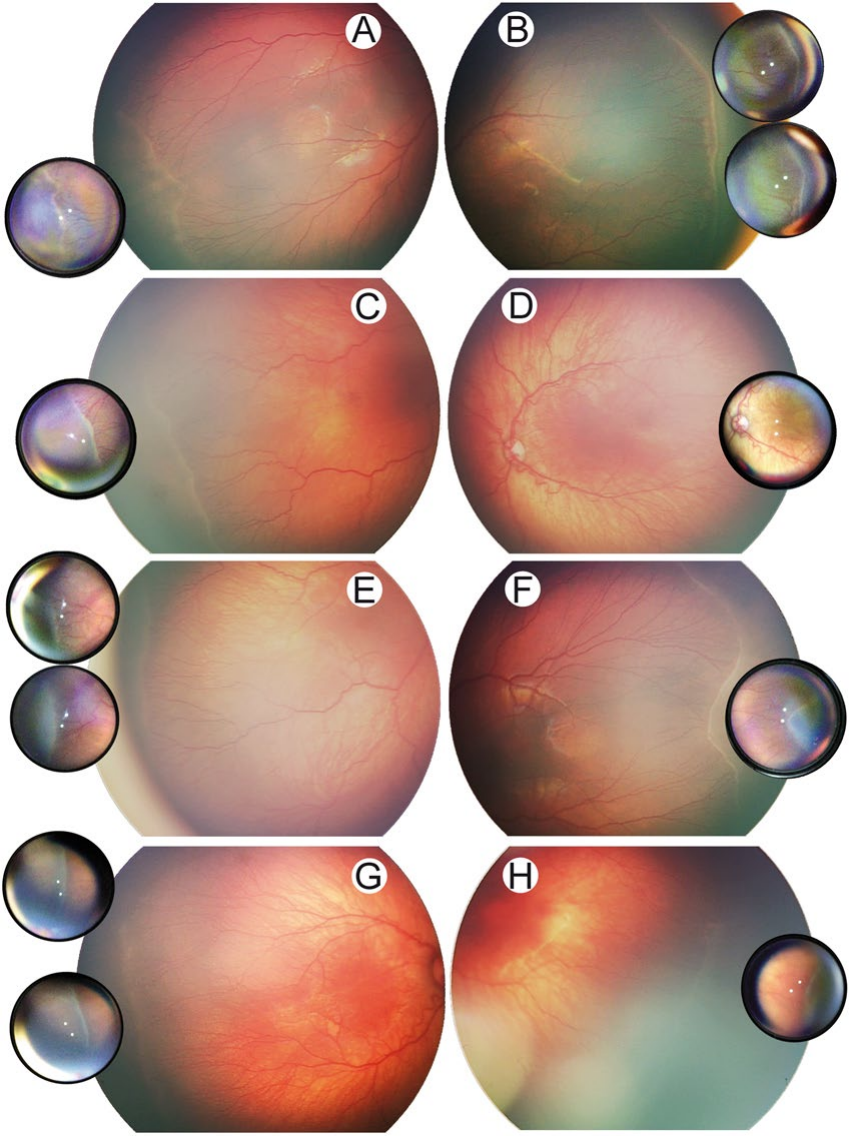
Comparison of retinopathy of prematurity documentation on smartphone-based fundus imaging and conventional fundus imaging. Conventional fundus imaging (large image sections) and smartphone-based fundus imaging (small juxtaposed image sections) were performed in the same eyes (**A–H**, each letter indicating one individual eye).

Comparison of SBFI with conventional fundus imaging revealed an equal and in some cases even higher level of detail on SBFI, potentially due to the higher image magnification (Figs. 2C, 3, and 4). Inter-rater reliability (squared Cohen’s kappa) for grading of plus disease and ROP stage on SBFI were 0.84 and 0.86, respectively. For conventional fundus imaging, these values were 0.76 and 0.90. Comparison with indirect ophthalmoscopy with regard to the grading of plus disease and ROP stage revealed a high agreement, sensitivity, specificity and Youden’s index, both for SBFI and conventional fundus imaging (Table 2). There was no statistically significant difference between sensitivities of SBFI and conventional fundus imaging (McNemar’s test for comparison of sensitivities: plus disease p = 0.41 and ROP p = 0.41). Determination of ROP zone was in agreement with ophthalmoscopy results in 80% of eyes for SBFI and in 82% of eyes for conventional fundus imaging.

**Table 2:** Agreement with evaluation by indirect ophthalmoscopy.

	Smartphone-based fundus imaging	Conventional fundus imaging
**Plus disease**		
Squared Cohen’s kappa	0.88	0.86
Sensitivity (any Plus disease)	0.90 (0.68–0.99)	0.80 (0.56–0.94)
Specificity (any Plus disease)	1.00 (0.89–1.00)	1.00 (0.89–1.00)
Youden’s index	0.90	0.80
**ROP zone**		
Squared Cohen’s kappa	0.43	0.53
**ROP stage**		
Squared Cohen’s kappa	0.81	0.93
Sensitivity (any ROP)	0.88 (0.68–0.97)	1.00 (0.86–1.00)
Specificity (any ROP)	0.93 (0.76–0.99)	0.96 (0.82–1.00)
Youden’s index	0.81	0.96

Data are kappa or sensitivities/specificities (95% confidence interval); ROP = retinopathy of prematurity.

Certainty of evaluation of plus disease on SBFI was not significantly different from evaluation on conventional fundus imaging (4.61 ± 0.87 vs. 4.79 ± 0.70, p = 0.26) as was certainty of evaluation of ROP stage (4.17 ± 1.42 vs. 4.19 ± 1.47, p = 0.95). Also, when considering only eyes with pre-plus/plus disease and ROP there were no statistically significant differences. Reasons for uncertainty in evaluation of plus disease and ROP are displayed in Table S1. The reason “overexposure” was never reported by any of the two raters.

## Discussion

As conventional digital fundus imaging systems for ROP are an enormous financial burden, particularly for health systems in middle- and low-resources settings, there is a huge need for novel and more cost-effective strategies for ROP screening^5,6,11^. SBFI is a less costly alternative to conventional imaging systems for ROP and may have the potential to facilitate telemedicine-based ROP screening in lower-resources settings. Especially if paramedical staff like optometrists, pediatric nurses or medical technicians are trained in SBFI, this may be a feasible solution. Both, the employment of paramedical staff to provide fundus examination for ROP screening and the use of telemedicine for remote ROP grading have already been successfully applied to conventional fundus imaging systems for ROP^9–11,13,15–17^.

Our study demonstrates that non-contact indirect SBFI represents a feasible alternative to conventional contact-dependent direct fundus imaging for ROP documentation and screening. Increased magnification allows for a higher level of image detail on SBFI, however results in a narrowed field-of-view. These results indicate that SBFI could provide a means to lower the financial burden of telemedicine-based ROP screening and documentation, in particular in economically disadvantaged regions.

So far only improvised techniques for indirect SBFI and few professional adapters have been tried in ROP^30–35^. Our study further supports existing data showing that SBFI is applicable to ROP. Furthermore, this is the first study to systematically compare the applicability of SBFI to ROP screening and documentation with an established conventional fundus imaging system for ROP. The level of detail is at least equal in SBFI compared with conventional fundus imaging. This might relate to a higher image magnification and, thus, a higher pixel density for the fundus’ solid angle imaged. While the RetCam resolves its field-of-view by 640 × 480 pixels, the iPod touch used for SBFI resolves its field-of-view by approximately 600 × 600 pixels (both in video mode). Due to the smaller field-of-view, examination of the periphery was more laborious with SBFI. As this could be compensated by a lens with a greater field-of-view, we also tested SBFI using a 40D lens. However, image quality was reduced and examination technique more challenging. Comparability of different SBFI devices in terms of image quality and learning curve for screening and documentation of ROP was not subject of our study and thus remains unclear^32–35^.

In contrast to conventional direct fundus imaging in ROP, indirect SBFI does not require contact with the corneal interface, and therefore represents a potentially less distressing imaging technique for the child and decreases the likelihood of infection. Another possible application of SBFI may be in combination with artificial intelligence (AI) for evaluation of plus disease and ROP on acquired single images and/or videos. The *Imaging and Informatics in Retinopathy of Prematurity (i-ROP) Research Consortium* has developed a deep learning algorithm for detection of plus disease and ROP on RetCam images which achieved promising results^39,40^. In contrast to conventional imaging techniques, SBFI would allow immediate online image transfer as well as local processing on the device which will be an advantage once AI solutions are more widely available.

The strength of this study is a comprehensive evaluation including field-of-view, image quality, examination time, agreement with grading by indirect ophthalmoscopy, and sensitivity and specificity for detection of plus disease and ROP stage. Furthermore, we compared against both an established conventional fundus imaging system for ROP as well as clinical examination by indirect funduscopy. Additional strengths are that the study population encompassed a variety of ROP manifestations and a wide range of gestational age and postmenstrual age at examination. Limitations of this study are a small sample size and the lack of quantitative assessment and comparison of image quality.

In conclusion, non-contact indirect SBFI is a feasible low-cost alternative to conventional contact-dependent direct fundus imaging for ROP screening and documentation. SBFI has the potential to considerably improve ROP care in middle- and low-resources settings. Future studies assessing the feasibility of SBFI for telemedicine-based ROP screening in these settings and its effects on the functional outcome of the screened infants are warranted.

## Supplementary information

Supplementary material

## Data Availability

The datasets generated and analyzed during the current study are available from the corresponding author on reasonable request.
