# Supplementary material for: Non-contact smartphone-based fundus imaging compared to conventional fundus imaging: a low-cost alternative for retinopathy of prematurity screening and documentation
Source: Sci Rep. 2019 Dec 23;9:19711. doi: 10.1038/s41598-019-56155-x (PMC6928229; doi:10.1038/s41598-019-56155-x)
Supplement: Supplementary file 1 — Supplementary material [file 41598_2019_56155_MOESM1_ESM.docx]

**Non-contact smartphone-based fundus imaging compared to conventional fundus imaging: a low-cost alternative for retinopathy of prematurity screening and documentation**

**Maximilian W. M. Wintergerst^1^, M.D., Michael Petrak^1^, M.D., Jeany Q. Li^1^, M.D., Petra P. Larsen^1^, M.D., Moritz Berger^2^, Ph.D., Frank G. Holz^1^, M.D., Robert P. Finger^1^, M.D., Ph.D., Tim U. Krohne^1*^, M.D.**

**Supplementary Table S1.** Reason for uncertainty in evaluation

|  | Reason for uncertainty in evaluation of (% and absolute number) | | | |
| --- | --- | --- | --- | --- |
|  | Plus disease | | ROP | |
|  | SBFI | CFI | SBFI | CFI |
| Fundus area examined too small | 20% (2) | 17% (1) | 40% (6) | 53% (8) |
| Image blurred | 10% (1) | 0% | 53% (8) | 40% (6) |
| Grading inconclusive albeit good image quality | 70% (7) | 83% (5) | 7% (1) | 7% (1) |

SBFI = smartphone-based fundus imaging; CFI = conventional fundus imaging
